# Mechanisms of mesenchymal stem/stromal cell function

**DOI:** 10.1186/s13287-016-0363-7

**Published:** 2016-08-31

**Authors:** Jeffrey L. Spees, Ryang Hwa Lee, Carl A. Gregory

**Affiliations:** 1 University of Vermont, Burlington, VT USA; 2 Institute for Regenerative Medicine, Texas A & M University College of Medicine, 206 Olsen Blvd., Room 228, MS1114, College Station, TX 77845 USA; 3 Department of Medicine, Stem Cell Core, University of Vermont, 208 South Park Drive, Ste 2, Colchester, VT 05446 USA

**Keywords:** Multipotent stromal cell, Mesenchymal stem cell, Paracrine, Tunneling nanotube, Mitochondria transfer, Microvesicle, Exosome

## Abstract

The past decade has seen an explosion of research directed toward better understanding of the mechanisms of mesenchymal stem/stromal cell (MSC) function during rescue and repair of injured organs and tissues. In addition to delineating cell–cell signaling and molecular controls for MSC differentiation, the field has made particular progress in defining several other mechanisms through which administered MSCs can promote tissue rescue/repair. These include: 1) paracrine activity that involves secretion of proteins/peptides and hormones; 2) transfer of mitochondria by way of tunneling nanotubes or microvesicles; and 3) transfer of exosomes or microvesicles containing RNA and other molecules. Improved understanding of MSC function holds great promise for the application of cell therapy and also for the development of powerful cell-derived therapeutics for regenerative medicine. Focusing on these three mechanisms, we discuss MSC-mediated effects on immune cell responses, cell survival, and fibrosis and review recent progress with MSC-based or MSC-derived therapeutics.

## Background

**Fig. 1:**
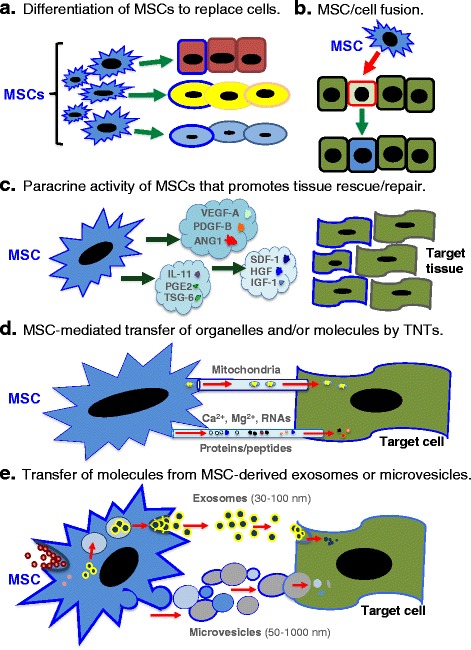
MSCs rescue and/or repair injured cells and tissues by diverse mechanisms. **a** Differentiation into replacement cell types. **b** Rescue of damaged or dying cells through cell fusion. **c** Secretion of paracrine factors such as growth factors, cytokines, and hormones. *VEGF* vascular endothelial growth factor, *PDGF* platelet-derived growth factor, *ANG1* angiopoietin-1, *IL-11* interleukin-11, *PGE2* prostaglandin E2, *TSG-6* TNF-stimulated gene-6, *SDF-1* stromal-derived factor-1, *HGF* hepatocyte growth factor, *IGF-1* insulin-like growth factor-1. **d** Transfer of organelles (e.g., mitochondria) and/or molecules through tunneling nanotubes (*TNTs*). *Ca* ^*2+*^ calcium, *Mg* ^*2+*^ magnesium. **e** MSC-mediated transfer of proteins/peptides, RNA, hormones, and/or chemicals by extracellular vesicles such as exosomes or microvesicles. Exosomes are generated through the endocytic pathway and released through exocytosis. By contrast, microvesicles are produced by cell surface budding and released directly from the plasma membrane. Note that the figure is not drawn to scale. Also, use of mechanisms **a**–**e** is not equivalent. For example, for MSCs administered intravenously, use of mechanism **c** is likely more relevant than are mechanisms (**a**) or (**b**)

Mesenchymal stem cells, also referred to as multipotent stromal cells or mesenchymal stromal cells (MSCs), have been the subject of intense scientific investigation since their initial discovery by Alexander Friedenstein in the late 1960s [1–5]. In their early studies, Friedenstein and colleagues demonstrated that MSCs, likely originating from the mesoderm, had the capacity to differentiate into a variety of mesenchymal tissue lineages such as osteoblasts, chondrocytes, and adipocytes. These observations sparked a substantial degree of interest in the potential application of MSCs for the repair of serious connective tissue trauma and disease [6–10]. It was originally hypothesized that, upon administration, MSCs would migrate to sites of injury, engraft, and differentiate into functional cells, resulting in regeneration of damaged or diseased connective tissues (Fig. 1a). Surprisingly, results from hundreds of animal studies and many human trials conducted over the past few decades have challenged this classic paradigm. In short, while MSCs were found to exhibit a remarkable degree of efficacy in a variety of disease models, it became increasingly apparent that the cells did not engraft in significant numbers or for durations sufficient to explain the results in terms of tissue replacement [11–15]. More surprisingly, MSCs were reported to engraft and differentiate into functional cells of tissues that did not originate from mesoderm [16, 17], questioning the long-established dogma that differentiation of adult stem cells is typically restricted to tissues derived from their germ layer of origin [18–20]. Later studies confirmed that the majority of results describing cross-germ line differentiation of MSCs could be ascribed to limitations in methodology or cell fusion events (Fig. 1b) [21–23]. Still largely unsolved, the mystery of efficacy without long-term engraftment, especially in non-mesodermal tissues, remains a source of considerable debate [24, 25]. In retrospect, a partial explanation for the benefits of MSC administration traces back to some of the very first observations made with bone marrow stromal cells. In the 1970s, Dexter and colleagues were the first to demonstrate that adherent stromal cells from bone marrow (later identified as MSCs) could sustain the growth, viability, and multipotent status of hematopoietic stem cells in long-term co-cultures that lacked growth factor supplementation [26–29]. Of particular interest was that the cultures achieved homeostasis with the self-renewal of progenitor cells balanced against the development of committed hematopoietic cells. These initial studies suggested that MSCs had the capacity to sustain the growth and viability of certain cell types through secretion of so-called trophic factors and even presented the notion that they could regulate certain facets of the immune system.

In an effort to reconcile discrepancies between the modest frequency and duration of engraftment with their remarkable healing properties, a contemporary view of MSC functionality is taking form. Rather than assuming long-term engraftment and differentiation, new hypotheses indicate that MSCs heal injured and diseased tissues/organs using alternative modes of rescue and repair that enhance cell viability and/or proliferation, reduce cell apoptosis, and, in some cases, modulate immune responses. The alternative modes of repair by MSCs include paracrine activity of secreted growth factors, cytokines, and hormones (Fig. 1c), cell–cell interactions mediated by tunneling nanotubes (TNTs; Fig. 1d), and release of extracellular vesicles (EVs) that contain reparative peptides/proteins, mRNA, and microRNAs (miRNAs; Fig. 1e). The purpose of this review is to examine and discuss key progress and important issues within this rapidly expanding area of regenerative medicine.

### Paracrine effects of administered MSCs

#### Immune modulation by MSCs

Some of the first evidence that MSCs could actively blunt immune responses originated from the results of mixed lymphocyte reaction (MLR) assays performed ex vivo [30–36]. These assays are based on the observation that T cells from preparations of immunologically mismatched peripheral blood mononuclear cells proliferate rapidly when mixed together under appropriate conditions [37, 38]. Results from MLR assays showed that T-cell expansion could be inhibited by the addition of MSCs to MLRs. While the majority of cell culture studies to date agree that such observations are mediated by MSC-derived soluble factors that do not cause T-cell apoptosis, several alternative mechanisms have also been proposed. Di Nicola et al. [31] employed a series of antibody blocking assays to implicate the role of transforming growth factor beta (TGFβ) and hepatocyte growth factor (HGF) whereas Aggarwal et al. [32] proposed a role for prostaglandin E2 (PGE2) based on their ability to ablate inhibitory responses with cyclooxygenase 2 (COX2) inhibitors. Aggarwal et al. further proposed that the secretion of PGE2 and related factors induced dendritic cells to up-regulate the anti-inflammatory cytokine interleukin (IL)10 while reducing the secretion of pro-inflammatory tumor necrosis factor alpha (TNFα) and IL12. This, in turn, initiates a shift in the ratio of T helper (T_h_) cells from a pro-inflammatory T_h_1 subtype to an anti-inflammatory T_h_2 subtype. This was accompanied by the differentiation of naive T cells to an immunoregulatory regulatory T cell (T_reg_) phenotype, thereby reducing the overall number of T_h_ cells. Similarly, Akiyama et al. [39] showed that MSCs could induce apoptosis of inflammatory T cells through activation of the Fas–Fas ligand axis. During this process, MSCs recruited additional T cells by secretion of monocyte chemotactic protein-1 (MCP-1) as part of a positive feedback loop. Apoptotic T-cell debris then activated phagocytes to secrete TGFβ, resulting in the differentiation of naive T cells into T_reg_ cells that can promote systemic immune tolerance [39]. In an alternative model, Meisel et al. [33] proposed an intriguing mechanism whereby MSC-derived indoleamine-2,3-dioxygenase (IDO) catalyzes the conversion of tryptophan to kynurenine in an interferon gamma-dependent manner. In turn, the kynurenine inhibits T-cell proliferation [40, 41]. This mechanism was later confirmed by utilizing the IDO antagonist 1-methyl-L-tryptophan [42]. In a series of experiments performed by Waterman et al. [43], it was reported that MSCs could be induced to express enhanced levels of IDO and PGE2 by transient stimulation of toll-like receptor (TLR)3 with polyinosinic-polycytidylic acid (poly I:C). MSC-mediated IDO activity has also been shown to enhance kidney allograft tolerance in mouse models through a mechanism involving T_reg_ up-regulation, demonstrating that IDO-mediated mechanisms of immune modulation can indeed occur in vivo [44]. Nitric oxide [45], galectin-1 and semaphorin-3A [46] have also been implicated as MSC-derived modulators of T-cell proliferation, but it is noteworthy to add that nitric oxide has only been shown to function as an MSC modulator in the murine system.

MSCs also have the capacity to modulate the activity of macrophages. This effect was initially described ex vivo using macrophage cultures stimulated with TLR ligands such as lipopolysaccharide (LPS), zymozan, or polyinosine-polycytidylic acid (poly I:C); these simulate the effects of bacterial or viral infection [47, 48]. When macrophages are challenged with such agents, they secrete inflammatory factors such as TNFα, IL1β, IL6, and reactive oxygen species. In the presence of MSCs, however, the ability of activated macrophages to secrete inflammatory factors was attenuated [32, 49]. Of interest, these observations were explained, in part, by MSC-mediated secretion of the extracellular protein TNFα-stimulated gene protein (TSG)6 [50]. In this model, exposure to zymozan caused cultured macrophages to secrete high levels of TNFα and other inflammatory mediators via the TLR2–nuclear factor kappa-B (NFkB) axis. TNFα activates TSG6 expression by MSCs and engages a negative feedback loop by inhibiting NFkB via activation of the CD44 receptor. Several in vivo studies have confirmed that MSC-derived TSG6 acts via the CD44 receptor to inhibit NFkB activity in macrophages, dendritic cells, and T_h_ cells in models of peritonitis [50], diabetes [51], and corneal transplant rejection [52]. In addition to the action(s) of TSG6, MSC-derived PGE2 has also been demonstrated to have potent effects on macrophages in vivo. In a murine model of sepsis, Nemeth et al. [53] demonstrated that, upon activation by LPS or TNFα, MSCs secreted PGE2. This caused the release of anti-inflammatory IL10 by macrophages and improved cell survival. Indeed, the role of PGE2 in MSC-mediated macrophage modulation is a common theme in many culture models [54, 55]. In an alternative mechanism proposed by Chen et al. [56], placental human MSCs inhibited the interaction of TLR4 with a key effector molecule, MyD88 [48], resulting in inhibition of secretory factors by macrophages. This process was inhibited by addition of a COX2 inhibitor, suggesting that the process was PGE2-dependent.

MSCs were reported to modulate the proliferation, differentiation, and immunoglobulin secretion of B cells without induction of apoptosis [57]. Transwell assays separating the two cell types but allowing for exchange of secreted factors showed that such MSC-mediated effects derived, in part, from the paracrine activity of soluble factors secreted by MSCs. These experimental results have since been replicated using purified B cells and unpurified preparations of peripheral blood mononuclear cells [58–60]; however, the paracrine mechanism was recently challenged by a co-culture study that suggested physical interaction between T cells and MSCs was necessary for MSCs to inhibit the activities of B cells [61]. Using a mouse model of allergy, Nemeth et al. [62] reported that MSC-derived TGFβ was critical in suppressing B-cell mediated allergic responses in vivo. They speculated that MSCs may recruit T_reg_ cells that down-regulate allergy-specific cytokine and immunoglobulin production as well as lung eosinophil infiltration. Consistent with their immune-modulatory properties, efficacy with MSC treatment has been demonstrated in a variety of inflammatory models of disease, including arthritis [63], Crohn’s disease [64], multiple sclerosis [65, 66], myocardial infarction [14], diabetes [51, 67], graft versus host disease [34, 68, 69], and corneal rejection [52].

#### Promotion of cell survival by MSCs

In addition to the paracrine effects of MSCs on immune cells, they also secrete a diverse repertoire of factors that support cell survival, including growth factors, cytokines, and extracellular matrix (ECM). Together, the components of the MSC secretome have the theoretical capacity to rescue injured cells, reduce tissue damage, and accelerate repair. This is exemplified by their natural roles as reticular cells that support the hematopoietic stem cell niche [26–28, 70, 71] and as vascular pericytes that support endothelial cells [72, 73]. The observation that MSCs can be isolated from a wide variety of tissues, such as bone marrow, adipose, ligament, skin, placenta, dental pulp, synovium, placenta, umbilical cord, and other fetal tissues [72, 74], lends support to the concept that they function endogenously as stromal support cells.

The pro-survival effect(s) of the MSC secretome on other cell types was first recognized through studies of long-term bone marrow cultures [26–29, 75] and embryonic cells [76]. Collectively, these cell culture studies provide for an attractive, paracrine-based explanation for the ability of MSCs to promote healing across a broad range of developmentally unrelated tissues and for myriad diseases and injury types. Detailed analysis of the MSC transcriptome and proteome has confirmed that they secrete a vast repertoire of paracrine pro-survival factors commonly referred to as trophic factors or mediators [77–82]. Of interest, the MSC-secreted factors comprise a diverse group of soluble peptides and proteins with complementary set(s) of biological activities that can accelerate progenitor cell self-renewal, stimulate angiogenesis, and minimize apoptosis and/or inflammation. Despite several decades of research and progress, the specific paracrine mechanisms by which administered MSCs improve cell survival and self-renewal under particular contexts of tissue rescue/repair remain largely undefined [75, 77].

In line with the traditional model of paracrine biology whereby cells secrete factors that regulate adjacent cells, it was initially thought that engrafted MSCs readily migrated into injured tissue and then remained to orchestrate repair. For many models of tissue injury, however, what was originally perceived as “MSC migration” turned out to be far less directed (e.g., non-specific, transient trapping of MSCs within the microvasculature and capillary network). Of particular interest, depending on their relative size (i.e., diameter), the majority of intravenously administered MSCs will typically lodge in the lung microvasculature upon the first pass through the circulation, regardless of the presence or absence of lung-specific injury. Notably, after intravenous MSC infusion, paracrine factors released into the blood by circulating MSCs or from trapped MSCs may indirectly influence survival signaling and the fate of distal cells previously compromised by injury or disease. Thus, for effect, paracrine factors produced by MSCs appear not to depend on long-term MSC engraftment, nor do they require the unlikely differentiation of mesodermal progenitors into tissues of ectodermal or endodermal lineages.

Some of the best evidence supporting an indirect role for MSCs in the repair of tissues/organs originates from studies of heart with infarction. In a rat model of myocardial infarction, MSCs modified with the gene encoding protein kinase B (a.k.a. Akt) engrafted into the myocardium, reduced pathological remodeling, and improved cardiac function [83]. The observed efficacy was later attributed to a paracrine effect mediated by secreted frizzled related protein (sFRP), a Wnt signaling inhibitor that reduces cardiomyocyte apoptosis [84–86]. Since these studies, a number of additional mechanisms for the paracrine action of MSC-derived factors on cardiac repair have been proposed, including secretion of angiogenic factors [87–89], stromal cell derived factor-1 (SDF-1) [90], and Jagged/Notch signaling [89, 91]. Of interest, MSC-mediated improvements in cardiac function could be achieved without long-term engraftment of MSCs [11]. Using a different approach, MSC-conditioned medium was employed to prime cardiac stem/progenitor cells prior to cardiac grafting in a rat model of myocardial infarction. The conditioned medium (CM) improved cardiac stem cell engraftment through mechanisms involving connective tissue growth factor and insulin signaling [92].

The role of MSCs in the protection of other damaged tissues has also been demonstrated. For example, intraperitoneally and intravenously administered MSCs from murine bone marrow and adipose tissue had a protective effect in a cisplatin-induced acute kidney injury (AKI) model [93], as evidenced by a reduction in the apoptosis of tubule cells and improved renal function. This effect appeared to be mediated by secreted factors since the results could be repeated by intraperitoneal administration of CM generated from the MSCs (MSC-CM). In contrast, Xing et al. [94] reported that murine MSC-CM containing HGF, vascular endothelial growth factor (VEGF)-A and insulin-like growth factor (IGF)-1 failed to protect the kidneys of mice against ischemia-reperfusion injury, whereas live MSCs had a significant protective effect. This is one of several examples in the field where apparently minor differences in the cell source, the culture conditions, duration of medium conditioning, and dosage can profoundly affect outcome. Such complexities have made elucidation of the mechanism(s) responsible for the protective effect of MSCs on kidney tissue challenging, but some progress has been made. For example, Zarjou et al. [95] demonstrated that the stress-responsive enzyme heme-oxygenase-1 (HO-1) played a role by utilizing MSC from bone marrow of HO-1^-/-^ mice. In this study, HO-1^+/+^ MSC-CM rescued pathology associated with cisplatin-induced AKI, while HO-1^-/-^ MSC-CM was ineffective. The authors attributed the difference in effect to enhanced levels of SDF-1, VEGF-A, and HGF in the HO-1^+/+^ MSCs. Indeed, immunological and transcriptional blocking experiments both confirm a protective role for VEGF-A [96–98] and IGF-1 [99] in mice with AKI and for VEGF-A in rats with cerebral ischemia (stroke) [100].

The utility of MSCs and their secreted products to protect cells and to foster tissue repair has been demonstrated in numerous efficacy-based studies across a broad range of tissue injury and disease models. While a comprehensive summary of the associated literature is beyond the scope of this review, some key examples of MSC-derived benefits include facilitation of wound healing [101], improved treatment of diabetes [102], enhancement of bone repair [103, 104], and effect(s) on cancer [105].

#### Effects of MSCs on fibrosis

Fibrosis is generally defined as a an accelerated accumulation of ECM factors (predominantly collagen type I) that prevents the regeneration of tissue. It can occur in virtually any tissue as a result of trauma, inflammation, immunological rejection, chemical toxicity, or oxidative stress. Current clinical strategies generally have poor outcomes in terms of efficacy and adverse effects [106]. Given the immunomodulatory and trophic properties of MSCs, they have become attractive candidates for the treatment of fibrosis and preclinical studies suggest they have a promising level of efficacy in a variety of models. While the anti-fibrotic effects of MSCs are likely to overlap with their anti-inflammatory and angiogenic properties, the specific mechanisms remain poorly understood. Nevertheless, a comprehensive review by Usuner et al. [107] suggests that their modes of action seem to fall under four categories: i) immune modulation, ii) inhibition of TGFβ-mediated differentiation of various cells types into ECM-secreting myofibroblasts by epithelial to mesenchymal transition, iii) inhibition of oxidative stress, and iv) matrix remodeling. For example, Ortiz et al. demonstrated that systemic murine MSC administration attenuated fibrosis in a bleomycin-induced lung injury model [108]. This was achieved through MSC-mediated secretion of IL1 receptor antagonist, which reduced infiltration of lymphocytes and neutrophils and their production of inflammatory and fibrotic mediators such as IL1 and TNFα. Using the same model, it was recently reported that MSCs had the capacity to inhibit fibrosis through the action of the secreted protein stanniocalcin-1 (STC-1) [109]. The authors demonstrated that STC-1 acted in multiple ways by reducing the secretion of collagen by fibroblasts, by reducing TGFβ output by endothelial cells and also through alleviating oxidative stress by uncoupling mitochondrial respiration via the induction of uncoupling protein 2. Using a model of chronic kidney injury, Huuskes et al. [110] demonstrated that MSCs improved kidney morphology and functionality when co-administered with the putatively anti-fibrotic hormone recombinant human relaxin (serelaxin). In this system, MSCs and serelaxin acted synergistically to reduce TGFβ-induced myofibroblast differentiation and collagen deposition while increasing the level of matrix metalloproteinase 2 (MMP2), a collagen-degrading enzyme.

### Transfer of mitochondria by TNTs and microvesicles

#### Discovery of TNTs

Rustom et al. [111] first reported TNTs as a communicating intercellular transport network formed in cultures of transformed cells (human 293 cells and rat PC12 cells) as well as primary cells from rat kidney. Endocytic organelles (lysosomes) and vesicles were shown to move through thin, 50–200 nm diameter filaments that stretched between cells. Incubation of cells in the inhibitor latrunculin B demonstrated a requirement for polymerized F-actin in TNT formation. Onfelt et al. [112] reported TNTs in human immune cells (e.g., natural killer cells, macrophages, and B cells) and later demonstrated that TNTs between macrophages had different properties and potentially differing functions; they observed thin filaments containing F-actin and also a thicker subset (0.7 microns) that contained both F-actin and microtubules. The thicker TNT subset was shown to transport mitochondria and lysosomal vesicles [113]. Other studies demonstrated that some TNTs were actinomyosin-dependent [114, 115]. For example, the Gerdes group showed that kidney cells treated with S-(-)-blebbistatin, a myosin II-specific inhibitor, increased the number of TNTs formed and also organelle transfer, whereas a general myosin inhibitor increased TNT number but significantly reduced organelle transfer [114].

#### Discovery of mitochondrial transfer by cultured MSCs

The first evidence that transfer of mitochondria might benefit injured target cells came from studies of human MSCs co-cultured with a unique lung epithelial cell line that lacked functional mitochondria (A549^rho^ cells) [116]. Utilizing a complementation screen to detect mitochondrial transfer and resulting cell growth, the Prockop group reported that human MSCs could restore aerobic respiration to A549^rho^ cells by transfer of mitochondria or mitochondrial DNA (mtDNA). Mitochondrial transfer from MSCs to rescued A549^rho^ cells was demonstrated by tracking genetic tags (i.e., mtDNA and nuclear DNA) and by time-lapse photomicroscopy of MSCs transduced with lentiviral vectors to target DsRed2 to mitochondria [116]. MSCs are now understood to transfer mitochondria to several different cell types, including epithelial cells, endothelial cells, and cardiac myocytes [117]. Such transfers are particularly evident when the potential target cells are injured or under stress. For example, MSCs were recently shown to prevent apoptosis in endothelial cells by transferring mitochondria during hypoxic/ischemic stress [118].

#### TNT formation and mitochondrial transfer in vivo

The first evidence that TNTs could form in vivo came from studies of the eye. Using wild-type, eGFP chimeric mice, and Cx3cr1(GFP) transgenic mice and confocal microscopy tracking, Chinnery et al. [119] documented membrane nanotubes that formed between bone marrow-derived MHC class II(+) cells in whole-mounted corneal tissue. Notably, they observed an increase in TNT frequency during corneal injury or inflammation. In a follow-up study with live imaging of myeloid cells in inflamed corneal explants from Cx3cr1(GFP) and CD11c(eYFP) transgenic mice, Seyed-Razavi et al. [120] showed de novo formation of nanotubes at a rate of 15.5 μm/min. These results demonstrated that TNTs could form in the absence of actual cell–cell contact and, furthermore, that they could then be directed from one cell toward another. Additional evidence for in vivo mitochondria or mtDNA transfer between cells came from studies of a remarkable canine transmissible venereal tumor that had persisted in feral dog populations for about 10,000 years. Rebbeck et al. [121] showed that the transmitted tumor cell line had obtained mitochondria (mtDNA) from multiple canine hosts over time. They suggested that fitness/persistence of canine transmissible venereal tumor benefited from the acquisition of host-derived mtDNA and through shedding of mutant and/or damaged mtDNA that could negatively impact mitochondrial biogenesis. Importantly, multiple research groups have shown that intercellular transfer of organelles and mtDNA is not limited only to the animal kingdom. Intercellular organelle trafficking and horizontal gene transfer in plants has been reported for both plastids [122] and mitochondria [123].

#### Proteins shown to control transfer of mitochondria by MSCs after tissue injury

Several recent studies have provided compelling evidence that administered MSCs can transfer mitochondria in vivo and, furthermore, that mitochondria transfer from MSCs can rescue injured pulmonary cells and ameliorate lung injury. Islam et al. [124] demonstrated that airway instillation of human MSCs could reduce LPS-mediated lung injury, in part, through transfer of mitochondria. Using live optical imaging, they documented transfer of vesicles containing labeled mitochondria from MSCs to alveolar epithelial cells that increased alveolar ATP levels and cell survival. Unlike wild-type MSCs, MSCs genetically modified for connexin 43 that were incapable of forming gap junctions and MSCs with dysfunctional mitochondria did not reduce acute lung injury [124].

Recent data from a cigarette smoke-induced model of lung injury suggest that donor source and age may affect repair by mitochondria transfer by MSC. Li et al. [125] found that transplantation of MSCs derived from induced pluripotent stem cells may provide enhanced repair after transplantation by virtue of increased TNT formation and mitochondria transfer relative to adult-derived MSCs.

Using loss- and gain-of-function approaches, Ahmad et al. [126] elegantly demonstrated that Miro-1, an outer mitochondrial membrane Rho-like GTPase, regulated the amount of mitochondrial transfer from MSCs to cultured lung epithelial cells. Enhanced expression of Miro-1 was shown to increase transfer of mitochondria from MSCs and treatment of mice with MSCs overexpressing Miro-1 reduced Rotenone lung injury and airway hyperresponsiveness and negative remodeling in several models of asthma [126].

#### Regulators of mitochondria transport identified in other cell types that may orchestrate mitochondrial transfer by MSCs

In addition to Miro-1, other proteins known to regulate intracellular mitochondrial dynamics (e.g., fusion, fission, tethering, and trafficking) [127, 128] may also promote or inhibit intercellular mitochondria transfer. Miro-1 and Miro-2 belong to a group of dynamin-related proteins that regulate mitochondrial division and fusion. They interact with TRAK1 and TRAK2 (identified as Milton in *Drosophila*), adaptor proteins that recruit kinesin motor proteins to mitochondria. The resulting adaptor–motor protein complex shuttles mitochondria along microtubules and was demonstrated to be critical for neuronal transport of mitochondria to axons, dendrites, and synapses [129–131]. Mitofusin 1 and 2 may also regulate mitochondria transfer as they are known to interact with Miro-1 and Miro-2 as well as TREK1/TREK2 in the adaptor–motor protein complex [132]. Perhaps not surprising, motor proteins are likely to be required for generation of some forms of TNTs. Myo-X (Myo10) is a myosin motor protein that localizes to the ends of cellular filapodia. It is unique in that it does not require substrate attachment to induce filapodia extension [133]. Co-culture studies in neuronal cells demonstrated that Myo10 was required for TNT formation from filapodia and overexpression of Myo10 resulted in increased TNT formation and vesicle transfer between cells [134].

Although the damage/injury signals that initiate mitochondrial transfer have yet to be identified, it is plausible that differences in intracellular Ca^+2^ or energy stores (e.g., glucose, ATP) may play a role in directing one cell to transfer mitochondria to another. For example, intracellular movement of mitochondria is highly sensitive to cytosolic Ca^+2^ levels. Wang and Schwartz [135] elegantly demonstrated that Ca^+2^ promotes Miro to interact with the motor domain of kinesin, thus blocking kinesin from the microtubule. Accordingly, mitochondria transfer from cell to cell may be affected by differences in intracellular Ca^+2^ concentration and/or localization. Consistent with this concept, TNTs have been shown to transfer Ca^2+^ and even electrical signals to neighboring cells through TNT-associated gap junctions [136, 137]. In addition, the level of available nutrients can alter movement of mitochondria. In neurons, Pekkurnaz et al. [138] reported that extracellular glucose and the enzyme O-GlcNAc transferase (OGT) affect mitochondrial motility by altering GlcNAcylation of Milton, an OGT substrate. As OGT activity is dependent on glucose, increased glucose was shown to decrease mitochondrial motility.

Of special interest, several reports indicate regulatory overlap or some form of integration between TNT formation and endosomal trafficking, as both interact with components of the exocyst complex that regulates vesicular transport from the Golgi apparatus to the plasma membrane [139, 140]. For example, Hase et al. [141] reported that M-sec, part of the exocyst complex, interacted with the small GTPase RalA and was required for TNT formation in a macrophage cell line. Furthermore, they showed that M-sec expression could induce cell protrusions de novo, some of which formed TNTs with adjacent cells. Subsequently, Schiller et al. [142] found that the transmembrane MHC class III protein leukocyte specific transcript 1 (LST1) was also required for TNT formation. At the cell membrane, LST1 was shown to interact with M-Sec, myosin, and myoferlin and also to recruit RalA, promoting its interaction with the exocyst complex [142]. Notably, some mechanisms (e.g., proteins) controlling TNT formation and/or mitochondrial transfer may be specific to specialized cell types such as neurons. However, in light of the conserved nature of intracellular adaptor/kinesin motor protein complexes, mitochondrial dynamics, and endosomal trafficking, it is probable that many mechanisms that control TNT formation and/or mitochondrial transfer are similar between many cell types, including MSCs.

#### Modifying mitochondrial transfer and/or mitochondria for clinical application

For future clinical application, harnessing mitochondrial transfer in a controlled and predictable manner will likely require further mechanistic insight. Importantly, recent advances in targeting of DNA to mitochondria may provide new tools to track or even perhaps to genetically alter mitochondria by modifying mtDNA as opposed to nuclear genes for proteins targeted to mitochondria (e.g., genes for mitochondrial membrane proteins). For example, Yu et al. [143] restored ATP synthesis in cells carrying mutant mtDNA for human NADH ubiquinone oxidoreductase subunit 4 (ND4) by infecting cells with an adeno-associated virus capsid (VP2) fused to a mitochondrial targeting sequence and the wild-type ND4 mitochondrial gene sequence. Following recent successful testing in non-human primates and human eyes ex vivo, the innovative method may soon be applied in clinical trials for treatment of Leber hereditary optic neuropathy, a disease caused by a mutation in the ND4 mitochondrial gene [144].

Despite the potential benefits of mitochondrial transfer or other TNT-mediated effects, it is worth noting that cell–cell communication by way of TNTs may also have some negative consequences. In contrast to their potential therapeutic benefits, TNTs also have potential to act as disease vectors for transmission of HIV/AIDS [145], bacteria [113], Prions [146], and oncogenic miRNAs [147].

### Transfer of RNAs and other molecules by EVs

The general term “extracellular vesicle” (EV) refers to membrane-bound vesicles released from most, if not all, somatic cell types (reviewed in [140, 148, 149]). Together, the EVs include exosomes, 30–100-nm plasma membrane-coated vesicles of endocytic origin; microvesicles, 50–1000-nm vesicles of non-endocytic origin; and apoptotic bodies, 1–5-μm vesicles released during membrane blebbing of apoptotic cells [150].

Cellullar exosomes are released when multivesicular bodies traffic to and fuse with the plama membrane in a regulated manner. Exosomes were first identified and isolated from cultures of normal and transformed cells during the 1980s [151–153]. Valadi et al. [154] made a key contribution when they demonstrated that both mRNA and miRNA could be exchanged between cells by virtue of exosomal transfer. Studying xenogenic co-cultures, they observed expression of various mouse proteins in human mast cells after exosomal transfer from murine cells, indicating successful translation of exosomally delivered mRNA into protein. As with exosomes isolated from diverse cell types, MSC-derived exosomes are reported to contain lipid raft domains [155] and tetraspanins known to alter the fusion state of cell membranes (e.g., CD9, CD81), Alix, a calcium-binding protein with roles in both endosomal trafficking and cell death, and TSG101, a tumor suppressor protein [156, 157]. Compared with exosomes, which are relatively homogenous upon release, microvesicles are heterogenous in both size and composition. Furthermore, regulatory mechanisms for microvesicular shedding from the membrane surface remain poorly understood.

Exosomes purified from MSCs have garnered tremendous interest in the field of regenerative medicine based on their ability to reduce apoptosis/necrosis in rodents after ischemic injury to the heart [158, 159], brain [160, 161], lung [162], liver [163], or kidney [164]. In addition, exosomal transfer from MSCs is reported to reduce inflammation and to increase cell proliferation during tissue repair [162, 165, 166]. Tomasoni et al. [167] showed that MSCs transferred exosomes with mRNA for IGF1R and IGF1 to cisplatin-damaged proximal tubular cells; this resulted in their expression of IGF1R, thereby increasing sensitization to IGF-1. The exosomal transfer improved renal cell survival and increased proliferation during repair after injury. In multiple drug-induced models of liver injury, treatment with MSC exosomes at the time of injury increased the number of proliferating cell nuclear antigen-positive proliferation cells while reducing the number of hepatocytes undergoing apoptotic cell death [168]. Treatment of a murine carbon tetrachloride-based injury model with exosomes from human umbilical cord-derived MSCs was shown to reduce liver fibrosis [169]. Following stroke in rats, treatment with MSC-derived exosomes was shown to promote angiogenesis, neurogenesis, neurite outgrowth, and recovery by virtue of transfer of miR-133b [170, 171]. In addition to RNAs, exosomes and microvesicles can deliver peptide/protein-based paracrine effectors such as growth factors, cytokines, and hormones. For example, transfer of Wnt4 by exosomes from human umbilical cord-derived MSCs improved repair of skin wounds in rats by altering cell proliferation [172].

Currently, many investigators and clinicians are interested in the potential of MSC-derived EV therapeutics for repair of injured and diseased tissue and to treat cancer [173, 174]. Most studies with exosome-based treatment of injured tissues/organs report positive outcomes, However, whether or not MSC-mediated transfer of exosomes, microvesicles, and/or their constituents promote or inhibit the activities of transformed cells in a way that would positively or negatively impact cancer remains context-dependent and controversial. For example, bone marrow MSCs were shown to reduce the growth of cultured breast cancer cells by transferring miR-127, -197, -222, and -223 through gap junctions and exosomes; these miRNAs are known to target CXCL12 (a.k.a. SDF-1) [175]. Lee et al. [176] suggested that exosomes from MSCs might suppress angiogenesis based on their containing miR-16, a miRNA that targets VEGF and was shown to reduce its expression in a breast cancer cell line. By contrast, Zhu et al. [177] reported that exosomes from human MSCs actually promoted tumor growth in vivo by inducing VEGF expression in tumor cells. Boelens et al. [178] reported cross-talk between stromal cells and breast cancer cells whereby stromal exosomes induced paracrine antiviral signals and stimulated juxtacrine Notch3 signaling that increased the number of therapy-resistant tumor-initiating cells. As with other paracrine effects of cell-based therapy or treatments based on administration of signaling agonists (e.g., growth factors), it is clear that care must be taken to avoid potential off-target treatment effects of administered EVs to avoid cancer cell propagation and/or metastasis.

Towards standardization of exosome-based therapy using MSCs or any cell type, identification of the most reliable and consistent vesicle isolation methods will be critical so that different laboratories can effectively compare their results. At present, several different methods of isolation are widely used, including centrifugation, filtration, immunoaffinity isolation with beads, and microfluidics. Notably, exosomes isolated from the same source by different methods may differ in amount and/or content [179–181].

Research aimed at improved understanding of mechanisms controlling cargo loading of exosomes will also be important. For protein-based cargo, Shen et al. [182] have reported some progress using expressed plasma membrane anchors. For miRNA-based cargo, Villarroya-Beltri et al. [183] recently identified specific miRNA sequence motifs that direct their loading into exosomes. Furthermore, they determined that sumoylated heterogenous nuclear ribonucleoprotein (hnRNPA2B1) was required for sorting of miRNAs into exosomes based on the specific motifs. Detailed characterization of MSC exosome content under various conditions and from all tissues will likely aid in a more predictable product in terms of therapy. For example, MSCs isolated from various tissues differ in terms of exosome content [184, 185] and MSCs from bone marrow with multiple myeloma were reported to differ in miRNA content relative to MSCs from control bone marrow [183].

## Conclusions

In light of promising results in animal models and patients, therapeutic use of MSCs and MSC-based products for treatment of tissue injury and disease is likely to undergo continued evaluation. As next steps, focusing efforts toward achieving standardized methods of MSC isolation, characterization, and administration has great potential to provide powerful new treatments with MSCs or MSC-derived products. In regard to the predominant mechanisms of MSC function, clarification of the relative role(s) that each mechanism plays during the rescue and repair of damaged tissues/organs following MSC administration may serve to improve treatment safety, efficacy, and predictability of outcome for patients.

## Abbreviations

CM: Conditioned Medium
COX2: Cyclooxygenase 2
ECM: Extracellular Matrix
EV: Extracellular Vesicle
HGF: Hepatocyte Growth Factor
HO-1: Heme-oxygenase-1
IDO: Indoleamine-2,3-dioxygenase
IGF: Insulin-like Growth Factor
IL: Interleukin
LPS: Lipopolysaccharide
miRNA: MicroRNA
MLR: Mixed Lymphocyte Reaction
MSC: Multipotent Stromal cell/Mesenchymal Stem Cell
mtDNA: Mitochondrial DNA
NFkB: Nuclear Factor Kappa-B
OGT: O-GlcNAc Transferase
PGE2: Prostaglandin E2
SDF-1: Stromal Cell-derived Factor-1
TGFβ: Transforming Growth Factor Beta
T_h_: T Helper
TLR: Toll-like Receptor
TNFα: Tumor Necrosis Factor Alpha
TNT: Tunneling Nanotube
T_reg_: Regulatory T Cell
TSG: TNF-stimulated Gene
VEGF: Vascular Endothelial Growth Factor
